# The effect of neonatal hypothyroidism and low family income on intellectual disability: A population-based cohort study

**DOI:** 10.1371/journal.pone.0205955

**Published:** 2018-11-07

**Authors:** Jin Young Nam, Young Choi, Mo Kyung Jung, Jaeyong Shin, Kyoung Hee Cho, Woorim Kim, Eun-Cheol Park

**Affiliations:** 1 Department of Public Health, Graduate School, Yonsei University, Seoul, Republic of Korea; 2 Institute of Health Services Research, Yonsei University College of Medicine, Seoul, Republic of Korea; 3 Department of Pediatrics, Bundang Cha Medical Center, Gyeonggi-do, Republic of Korea; 4 Department of Preventive Medicine, Yonsei University College of Medicine, Seoul, Republic of Korea; Universidade do Porto Faculdade de Medicina, PORTUGAL

## Abstract

**Background:**

To investigate relationships among neonatal hypothyroidism, family income, and intellectual disability, as well as the combined effects of neonatal hypothyroidism and low family income on intellectual disability.

**Methods:**

Data were extracted from the National Health Insurance Service-National Sample Cohort from 2002 to 2011. This retrospective study included 91,247 infants. The presence of intellectual disability was based on the disability evaluation system in Korea. Newborn hypothyroidism was identified from diagnosis and prescription codes. Family income was determined from average monthly insurance premiums. Cox proportional hazards models were used to calculate adjusted hazard ratios.

**Results:**

Of the 91,247 infants, 208 were considered to have intellectual disability (29.18 cases per 100,000 person-year). The risk of intellectual disability was higher in infants with hypothyroidism than in those without hypothyroidism (hazard ratio = 5.28, *P*: < .0001). The risk of intellectual disability was higher in infants with low family income than in those with high family income (hazard ratio = 2.32, *P*: < .0001). The risk of intellectual disability was higher in infants with hypothyroidism and low family income than in those without hypothyroidism and with high family income (hazard ratio = 36.05, *P*: < .0001).

**Conclusions:**

Neonatal hypothyroidism and low family income were associated with the risk of intellectual disability in Korea. Additionally, neonatal hypothyroidism and low family income significantly increased the risk of intellectual disability. Public health policymakers should consider providing additional resources for alleviating neonatal hypothyroidism among low-income families.

## Introduction

Intellectual disability (ID) refers to a significant reduction in the ability to understand, learn, and apply adaptive skills, and appears before 18 years of age [1, 2]. The recent definition of ID proposed by the American Association on Intellectual and Development Disability reflects both low intellectual functioning (an IQ test score of 70 to 75 or less) and poor adaptive behavior, which involves limitations in conceptual, social, and practice skills in daily life [2]. Previous studies have reported different causes of ID, such as genetic conditions, problems during pregnancy or birth (congenital hypothyroidism [CHT] and fetal alcohol syndrome), birth defects that affect the brain (asphyxia), problems during infancy and childhood (serious head injury or meningitis) [2–4], and socioeconomic status (SES) [5–7]. Although the causes of ID are not documented in more than half of the children with ID, preventable causes of ID, such as neonatal hypothyroidism (NH), are noted in many children [8, 9].

Neonatal hypothyroidism is well known as one of the most common preventable causes of ID, and hypothyroidism can be prevented with early detection and appropriate treatment soon after birth in the first 2–6 postnatal weeks [4, 10]. Studies have shown that IQs are lower in children with NH who are not adequately treated in the first 2–3 years after birth, compared to unaffected children [11, 12]. Unfortunately, the clinical signs of NH are not obvious until a later age [13]; therefore, early detection through infant screening programs and adequate treatment during early childhood are important to prevent ID. There are several causes of newborn hypothyroidism, including thyroid gland dysgenesis or ectopic location, exposure to iodides, TSH deficiency, TRH deficiency, inborn defect in hormone synthesis or effects, and maternal goitrogen ingestion [14]. Therefore, maternal health conditions might significantly influence fetal or NH.

Childhood SES has been reported to be associated with cognitive ability measured by IQ [5]. Studies have shown that children living in households with low income or in poverty are more likely to have ID [15] and to obtain lower scores on standardized tests of academic achievement [7, 16]. In addition, low- or middle-income countries have been found to have higher prevalences of ID in children/adolescents, compared to high-income countries [17]. A previous study indicated that there is a causal effect for parental financial resources on cognitive performance in children [18], and another study reported an association between the development of specific areas of the brain and poverty [19]. Furthermore, previous studies have mentioned that maternal conditions might be related to socioeconomic factors [8, 20], and inverse socioeconomic differences have been found to be associated with ID [9, 21, 22]. Additionally, a correlation has been reported between an increase in the risk of ID in children and the presence of unhealthy conditions in mothers during pregnancy in disadvantaged populations [8]. However, there is no information on the combined effect of NH and family income on ID in early childhood.

The present study investigated the relationships among NH, family income, and ID, as well as the combined effect of NH and low family income on ID.

## Materials and methods

### Data collection and participants

#### Data source

The National Health Insurance Service–National Sample Cohort (NHIS-NSC) is a population-based cohort established by the National Health Insurance Service (NHIS) in South Korea [23]. Korea supports universal health insurance for all citizens via a single-insurer system [23]. The purpose of this cohort is to provide public health researchers and policymakers with representative, useful information on the utilization of health insurance and health examinations among Korean citizens [23]. The NHIS provides benefits for prevention, diagnosis, disease and injury treatment, rehabilitation, births, deaths, health promotion, and national records for healthcare utilization and prescriptions [23]. The NHIS-NSC was designed to include a target population of 46 605 433 individuals from 47 851 928 individuals included in the 2002 NHIS, excluding non-citizens and special purpose employees with an unidentifiable income level [23]. From the target population, a representative sample cohort was selected. The cohort included 1 025 340 participants, who were randomly selected. This comprised 2.2% of the total eligible Korean population in 2002, and the participants were followed for 11 years until 2013, unless a participant’s eligibility was disqualified because of death or emigration [23]. Systematic stratified random sampling with proportional allocation within each stratum was performed using the individual’s total annual medical expenses, including age, sex, residence, and health insurance type, as a target variable for sampling [24]. Therefore, the representativeness of the sample was evaluated by examining whether a 95% confidence interval for the sample’s average total annual medical expenses included the population average and whether the finding was satisfied for every stratum [23]. The cohort sampled in the 2002 NHIS database was followed until 2013. Its participants were still eligible for health insurance. The number of infants (age 0) in the initial cohort and those added annually are provided in the table [23]. In addition, the structure of the cohort data was semi-dynamic, and approximately 9,000 newborn infants were added using stratified random sampling every year (the same sampling method as in the first wave) to preserve the national representativeness of the original sample by replacing individuals lost owing to death over time. The unique de-identified numbers of the patients, age, sex, types of insurance, diagnoses according to the International Classification of Diseases (ICD-10), medical costs, procedures, and prescribed drugs were included. The degree of ID was evaluated using the disability evaluation system in accordance with the welfare enforcement regulation for disabled persons in Korea. In addition, unique de-identified numbers were linked to mortality information from the Korean National Statistical Office. Currently, the NHIS plans to maintain regular annual cohort updates for the NHIS-NSC [23].

#### Data collection

We performed a cohort study of newborn infants between 2002 and 2011. Among 1,025,340 NHIS-NSC enrollees in 2002, we selected 9,562 newborn infants. Additionally, we selected 81,685 newborn infants included between 2003 and 2011. To ensure confidentiality, the NHIS-NSC included only the birth year and not the exact birth date. Therefore, the coded infant birth status did not always appear within the database. This study adhered to the tenets of the Declaration of Helsinki, and the study design was reviewed and approved by the ethics board of the Graduate School of Public Health in Yonsei University (2-1040939-AB-N-01-2016-332).

### Intellectual disability and follow up

The outcome variable for this study was ID. The occurrence of ID was based on the disability evaluation system. According to this system, children with ID should be diagnosed by a psychiatrist, neurologist, or rehabilitation specialist in a hospital. In order to assess ID, specialists commonly use the Bayley Scales of Infant Development (for early childhood), Stanford-Binet Intelligence Scales (2–23 years), K-Wechsler Preschool and Primary Scale of Intelligence (3–7 years), and Korean Educational Development Institute-Wechsler Intelligence Scale for Children (5–15 years). The specialists estimate intellectual functioning skills and adaptive behavior through these scales and determine the IQ score [25]. All infants born between 2002 and 2011 were observed from birth until December 31, 2013 or until registration of disability or death, whichever came first.

### Neonatal hypothyroidism

Neonatal hypothyroidism was identified from diagnosis and prescription drug codes. We considered an infant to have hypothyroidism if the infant had at least one outpatient claim for hypothyroidism (ICD-10 codes E00, E01, E02, and E03) and one or more filled prescriptions for thyroid hormone therapy (levothyroxine) during the first year of life.

### Family income as part of the socioeconomic status

We used national health insurance (NHI) premiums as a proxy for family income. In Korea, individuals qualify for medical aid if their family income is less than $600 per month. If family income is more than $600 per month, individuals qualify for NHI. The NHI premiums are mandatory and imposed based on monthly salary, taxable income, and assets. Individuals who qualified for NHI were distributed between the 1st and 100th percentiles for income, while those who qualified for medical aid were classified as the zero percentile. We classified family income as follows: (1) low income (medical aid/below the 40th percentile), (2) mid-level income (41st–70th percentile), and (3) high income (71st–100th percentile). Family income referred to household income during the infants’ first year of life.

### Covariates

Demographic factors (sex, residence area, year of birth) were included. Low birth weight (<2500 g) and preterm birth (<37 weeks) were identified from medical records (ICD-10 code P07). Birth asphyxia was identified from medical records as intrauterine hypoxia or birth asphyxia (ICD-10 codes P20 and P21). Congenital factors were identified from the medical records. Congenital malformations in the following systems/organs were included: nervous system (codes Q00, Q01, Q02, Q03, and Q04); eye, ear, face, and neck (codes Q1.x); circulatory system (codes Q2.x); respiratory system (codes Q30, Q31, Q32, Q33, and Q34); digestive system (codes Q38, Q39, Q40, Q41, Q42, Q43, Q44, and Q45); genital organs (codes Q5); urinary system (codes Q60, Q61, Q62, Q63, and Q64); musculoskeletal system (codes Q65, Q66, Q67, Q68, Q69, and Q7); and others (codes Q8). Additionally, cleft lip and cleft palate (codes Q35, Q36, and Q37) were included. Chromosomal abnormalities were identified according to the ICD-10 code Q9. Inborn errors of metabolism were identified according to the ICD-10 codes E7, E80, E83, E84, E85, E87, E88, E89, and E9. These codes were obtained for all cohort members from the NHIS-NSC.

### Statistical analysis

We calculated the distribution of general characteristics of the study participants born between 2002 and 2011. The relationships among hypothyroidism, family income, and ID were analyzed using time-to-event methods. Cumulative incidence curves were generated for comparison of unadjusted ID rates according to hypothyroidism and family income groups. In order to examine whether ID is related with children with hypothyroidism or with low income, multivariable analyses were performed using Cox proportional hazards models to calculate adjusted hazard ratios (HRs) (95% confidence intervals [CIs]) as an estimate of the relative rate of ID. The proportionality assumption was tested by plotting Schoenfeld-like residuals. In addition, a combined Cox proportional hazards model was planned if an interaction between hypothyroidism and family income was found to affect ID. We performed a sensitivity analysis excluding trisomies including Down syndrome, Edwards’ syndrome, and other tisomies (ICD 10: Q90, Q91, Q92). All statistical analyses were performed using SAS 9.4 (SAS Institute, Inc., Cary, NC, USA). The level of significance was set at *P* < 0.05.

## Results

Table 1 presents the general characteristics of the study population according to NH or family income. Of the 91,247 infants included in this study, 124 were found to have NH and 19,942 were found to have low family income. Among the infants with NH, ID was noted in 8.87%, and 0.22% of those without NH had ID. In regards to family income, 0.38%, 0.21%, and 0.15% of infants with low-, middle-, and high family income, respectively, had ID.

**Table 1:** General participant characteristics according to neonatal hypothyroidism or family income from 2002 to 2011.

		Total		Neonatal hypothyroidism				Family income					
				Yes (n = 124)		No (n = 91123)		Low (n = 19942)		Middle (n = 39152)		High (n = 32153)	
		N	(%)	N	(%)	N	(%)	N	(%)	N	(%)	N	(%)
Intellectual disability													
	No	91039	(99.77)	113	(91.13)	90926	(99.78)	19866	(99.62)	39069	(99.79)	32104	(99.85)
	Yes	208	(0.23)	11	(8.87)	197	(0.22)	76	(0.38)	83	(0.21)	49	(0.15)
Hypothyroidism													
	No	91123	(99.86)	-		-		19920	(99.89)	39101	(99.87)	32102	(99.84)
	Yes	124	(0.14)	-		-		22	(0.11)	51	(0.13)	51	(0.16)
Household income													
	Q1(Low)	19942	(21.85)	22	(17.74)	19920	(21.86)	-		-		-	
	Q2	39152	(42.91)	51	(41.13)	39101	(42.91)	-		-		-	
	Q3(High)	32153	(35.24)	51	(41.13)	32102	(35.23)	-		-		-	
Sex													
	Man	47212	(51.74)	53	(42.74)	47159	(51.75)	10276	(51.53)	20230	(51.67)	16706	(51.96)
	Women	44035	(48.26)	71	(57.26)	43964	(48.25)	9666	(48.47)	18922	(48.33)	15447	(48.04)
Residence													
	Rural	27213	(29.82)	31	(25.00)	27182	(29.83)	7314	(36.68)	11931	(30.47)	7968	(24.78)
	Urban	64034	(70.18)	93	(75.00)	63941	(70.17)	12628	(63.32)	27221	(69.53)	24185	(75.22)
Low birth weight(<2500g)													
	No	90059	(98.70)	102	(82.26)	89957	(98.72)	19657	(98.57)	38676	(98.78)	31726	(98.67)
	Yes	1188	(1.30)	22	(17.74)	1166	(1.28)	285	(1.43)	476	(1.22)	427	(1.33)
Birth asphyxia													
	No	91135	(99.88)	123	(99.19)	91012	(99.88)	19910	(99.84)	39107	(99.89)	32118	(99.89)
	Yes	112	(0.12)	1	(0.81)	111	(0.12)	32	(0.16)	45	(0.11)	35	(0.11)
Congenital malformations*													
	0	80598	(88.33)	87	(70.16)	80511	(88.35)	17683	(88.67)	34625	(88.44)	28290	(87.99)
	1	9573	(10.49)	25	(20.16)	9548	(10.48)	2022	(10.14)	4079	(10.42)	3472	(10.80)
	2+	1076	(1.18)	12	(9.68)	1064	(1.17)	237	(1.19)	448	(1.14)	391	(1.22)
Chromosomal abnormalities													
	No	91080	(99.82)	112	(90.32)	90968	(99.83)	19908	(99.83)	39074	(99.80)	32098	(99.83)
	Yes	167	(0.18)	12	(9.68)	155	(0.17)	34	(0.17)	78	(0.20)	55	(0.17)
Inborn error of metabolism													
	No	90755	(99.46)	120	(99.46)	90635	(96.77)	19826	(99.42)	38947	(99.48)	31982	(99.47)
	Yes	492	(0.54)	4	(0.54)	488	(3.23)	116	(0.58)	205	(0.52)	171	(0.53)
Year of birth													
	2002	9562	(10.48)	17	(10.47)	9545	(13.71)	2233	(11.20)	4274	(10.92)	3055	(9.50)
	2003	9437	(10.34)	8	(10.35)	9429	(6.45)	2072	(10.39)	4255	(10.87)	3110	(9.67)
	2004	9320	(10.21)	8	(10.22)	9312	(6.45)	2047	(10.26)	4153	(10.61)	3120	(9.70)
	2005	8556	(9.38)	9	(9.38)	8547	(7.26)	1910	(9.58)	3765	(9.62)	2881	(8.96)
	2006	7872	(8.63)	7	(8.63)	7865	(5.65)	1695	(8.50)	3400	(8.68)	2777	(8.64)
	2007	9766	(10.70)	10	(10.71)	9756	(8.06)	2151	(10.79)	4181	(10.68)	3434	(10.68)
	2008	9392	(10.29)	21	(10.28)	9371	(16.94)	2041	(10.23)	3897	(9.95)	3454	(10.74)
	2009	8616	(9.44)	17	(9.44)	8599	(13.71)	1797	(9.01)	3579	(9.14)	3240	(10.08)
	2010	9032	(9.90)	9	(9.90)	9023	(7.26)	1886	(9.46)	3725	(9.51)	3421	(10.64)
	2011	9694	(10.62)	18	(10.62)	9676	(14.52)	2110	(10.58)	3923	(10.02)	3661	(11.39)

*Congenital malformations of the nervous system, eye, ear, face, neck, circulatory system, respiratory system, genital organs, urinary system, digestive system, musculoskeletal system, and other organs/systems, as well as cleft lip and cleft palate

**Fig 1:**
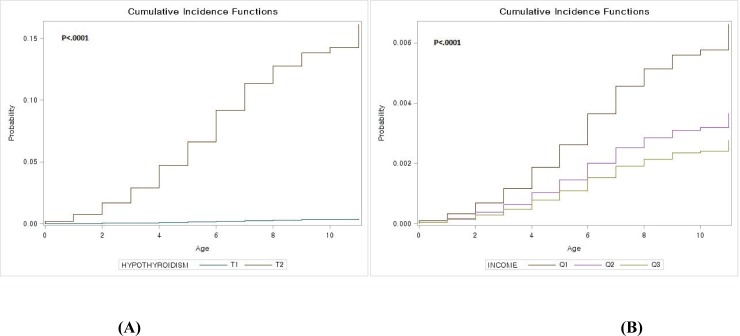
Cumulative incidence curves for the occurrence of intellectual disability according to neonatal hypothyroidism or family income. (A) Cumulative incidence of intellectual disability according to neonatal hypothyroidism. (B) Cumulative incidence of intellectual disability according to family income. T1, with neonatal hypothyroidism; T2, without neonatal hypothyroidism; Q1, low family income; Q2, middle family income; Q3, high family income.

Fig 1 presents the cumulative incidence of ID according to the NH and family income groups. The incidence of ID was significantly higher in infants with NH than in those without NH (*P* < 0.0001). Additionally, the incidence of ID was significantly higher in infants with low family income than in those with high family income (*P* < 0.0001).

Table 2 presents the results of the Cox proportional hazards analysis. The risk of ID was higher in infants with NH than in those without NH (HR = 5.28, 95%CI: 2.65–10.52). Additionally, infants with low family income were likely to have a high risk of ID. There was a statistically significant association between low family income and ID in infants (HR = 2.32, 95%CI: 1.61–3.34).

**Table 2:** Cox proportional hazards analysis for associations between the presence of intellectual disability and risk factors.

			Intellectual disability			
		Person-year	N	HR	95% CI	
Hypothyroidism						
	No	711772	197	1.00		
	Yes	903	11	5.85	(2.93-	11.69)
Household Income						
	Q1(Low)	155889	76	2.32	(1.61-	3.34)
	Q2	308284	83	1.24	(0.87-	1.78)
	Q3(High)	248502	49	1.00		
Sex						
	Man	369241	143	1.83	(1.36-	2.46)
	Women	343434	65	1.00		
Residence						
	Rural	212600	82	1.43	(1.08-	1.90)
	Urban	500075	126	1.00		
Low birth weight (< 2500g)						
	No	704134	196	1.00		
	Yes	8541	12	2.15	(1.16-	3.97)
Birth asphyxia						
	No	711834	207	1.00		
	Yes	841	1	1.32	(0.18-	9.95)
Congenital malformations*						
	0	630188	106	1.00		
	1	74483	67	4.13	(3.01-	5.68)
	2+	8004	35	8.97	(5.66-	14.20)
Chromosomal abnormalities						
	No	711551	169	1.00		
	Yes	1124	39	40.67	(26.04-	63.51)
Inborn error of metabolism						
	No	708613	201	1.00		
	Yes	4062	7	2.64	(1.22-	5.73)
Year of birth						
	2002	114496	50	1.00		
	2003	103712	32	0.72	(0.46-	1.14)
	2004	93132	32	0.75	(0.47-	1.18)
	2005	76914	27	0.70	(0.43-	1.15)
	2006	62956	17	0.61	(0.35-	1.09)
	2007	68352	18	0.59	(0.33-	1.03)
	2008	56338	18	0.83	(0.47-	1.48)
	2009	43074	5	0.32	(0.12-	0.83)
	2010	36121	6	0.56	(0.23-	1.38)
	2011	29080	3	0.47	(0.14-	1.59)

HR, hazard ratio; CI, confidence interval

*Congenital malformations of the nervous system, eye, ear, face, neck, circulatory system, respiratory system, genital organs, urinary system, digestive system, musculoskeletal system, and other organs/systems, as well as cleft lip and cleft palate

**Fig 2:**
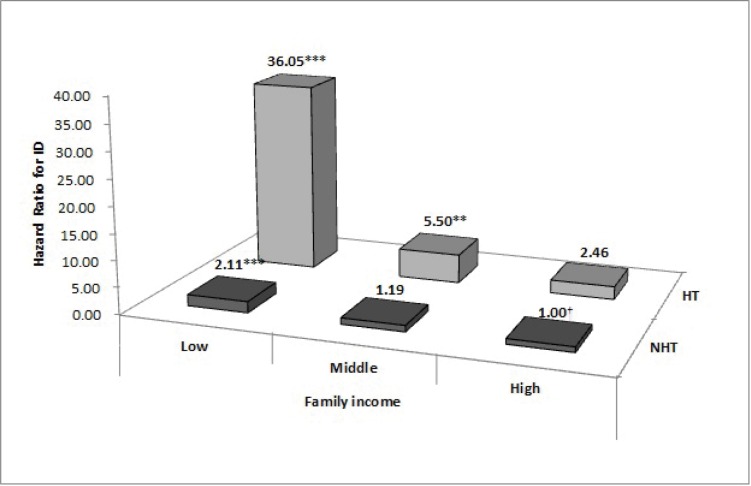
Combined effect of neonatal hypothyroidism and family income on intellectual disability. Adjusted for sex, residence area, low birth weight, birth asphyxia, congenital malformations, chromosomal abnormalities, and inborn errors of metabolism. ID, intellectual disability; NH, neonatal hypothyroidism; No NH, no neonatal hypothyroidism. *P < 0.05, **P < 0.01, ***P < 0.001. †reference.

Fig 2 presents the results of the Cox proportional hazards analysis of the combined effect of NH and family income on ID. Infants with NH and low or mid-level family income were more likely to have a high risk of ID than those without NH and high family income (low: HR = 36.05, 95%CI: 12.79–101.61; mid-level: HR = 5.50, 95%CI: 1.82–16.61). In addition, infants with low family income showed a high risk of ID irrespective of NH, compared with the reference group (low family income without NH; HR = 2.11, 95%CI: 1.46–3.07) (See S1 Table, available online).

S2–S4 Tables present the result of sensitivity analysis excluding trisomes such as Down syndrome. After excluding these children, infant with NH was high risk of ID compared with those without NH (HR = 7.86, 95% CI: 2.70–22.89). In addition, infants with the lowest family income had a high risk of ID compared with those with the highest family income (HR = 2.39, 95% CI: 1.62–3.52) (See S3 Table, available online).

## Discussion

The present study found that NH increases the risk of ID. Additionally, low family income was associated with a high risk of ID. Moreover, we noted a significant combined effect for low family income and NH on the risk of ID. To our knowledge, this population-based cohort study is the first to examine the associations among NH, family income, and ID in infants born between 2002 and 2011.

Our finding of an association between NH and ID is consistent with the findings of previous studies, which showed that NH is a common, preventable cause of ID [10, 26, 27]. In the study by Grosse et al., among children with clinically diagnosed CHT, 8–28% were found to have ID [26]. In the present study, 8.8% of infants with NH had ID during childhood, and the risk of ID was approximately six-fold higher in these infants with NH than in those without NH.

Previous studies have assessed SES, particularly poverty and low family income, in relation to ID. In a meta-analysis, Maulik and colleagues demonstrated that low- and middle-income countries had a higher prevalence of ID that was almost twice that of high-income level countries [17]. In Emerson’s study, children with ID were more likely to have mothers with low educational level, low weekly household income, and poverty level, compared with children without ID [15]. Furthermore, Hair and colleagues mentioned that children born into poor families showed low brain development [7], and Hackman and colleagues reported that low SES in childhood was associated with poor cognitive development and that this was positively related to intelligence and academic achievement from early childhood through adolescence [5, 6]. The present study showed that the risk of ID was over 2.3-fold higher in infants born into low-income families than in infants born into high-income families. Therefore, our findings are consistent with previous findings.

The combination of NH and low family income was strongly associated with a high risk of ID in infants. Moreover, we found that infants with low family income had a high risk of ID irrespective of NH. These findings indicate that NH is a strong risk factor for ID, especially infants with NH, and that low family income is significantly associated with the risk of ID.

The effect of NH and low family income might be explained by several mechanisms. First, NH is one of the most easily preventable causes of ID. If newborns with NH are treated adequately and promptly within the first 2–6 postnatal weeks, they can grow and develop normally [28]. However, if children remain untreated or their clinical symptoms become evident in the second half of the first year of life, delay in the development of motor skills and ID are unavoidable [28]. Moreover, IQs have been reported to be lower in children who are treated inadequately in the first 2–3 years after birth than in unaffected children [10, 11]. Unfortunately, the majority of newborns with NH do not have obvious manifestations of hypothyroidism, as some residual thyroid function might be present, and the clinical symptoms and signs of hypothyroidism are relatively nonspecific, making clinical diagnosis difficult [27]. The best approach to detect infants with NH is newborn screening programs, and such programs are being performed in many developed countries to prevent ID [27]. Second, SES, especially poverty, has been shown to affect brain development in infants [5–7, 13, 29]. In the study by Hackman et al., prenatal factors, parental care, and cognitive stimulation were suggested to be potential factors that influence the effect of SES on neurocognitive development [5]. Other studies have shown that maternal inverse SES is related to ID in infants, because maternal conditions might be associated with SES factors [8, 20]. Additionally, low SES in pregnant women might be associated with high levels of stress, malnutrition, and high infection rates during pregnancy [5], as well as birth-related infections and injuries due to poor maternal and child health care facilities [30], which could increase the likelihood of premature birth, impaired fetal growth [31], and intra-uterine growth retardation [30]. In particular, thyroid deficiency or disease during pregnancy has been found to increase the risk of low IQ or encephalopathy in newborns [30, 32, 33], and women with less strictly managed thyroid disease have been deemed more likely to have affected infants [33]. In order to prevent ID in children, it is important to maintain serum free T4 levels in the mid-normal range and TSH levels in the normal range [10]. To achieve treatment goals, serum free T4 and TSH levels should be monitored periodically, and oral T4 (levothyroxine) doses should be appropriately managed [10]. The exact dose volume, time to dose, and feeding method for infants should be determined, and food intake should be avoided during the administration period [27]. Meanwhile, low SES of the mother might be associated with low education, low income, low health insurance coverage, and high depression [29, 34]. Also, low SES infants are more likely to suffer from high blood lead levels, iron deficiency, stunting, and sensory impairment, and these outcomes likely reflect conditions associated with low SES, including inadequate nutrition, insufficient access to health care, failure to get recommended immunization, and exposure to tobacco smoke [35]. Therefore, these conditions might increase the risk of ID.

The present study has several strengths. This study included a large population of infants and implemented a population-based design. Data were obtained from the nationally representative NHIS-NSC. Additionally, we used the unique personal identity number of each Korean resident and linked it to national insurance and mortality data for follow-up. This study provided evidence of the associations of NH and low family income with ID, as well as the significant effect of NH and low family income on the occurrence of ID. Cumulative evidence has explained the association between socioeconomic disadvantage and ID. In addition, we used family income to measure objective method. Nevertheless, few longitudinal studies have been performed, and previous studies had issues with regard to the measurement of ID [36]. In our study, the measurement of ID was comparatively reliable and was considered valid because it was based on the definition of ID according to law, which considers medical opinion and various examinations by physicians. Our findings reinforce the value of analyzing different measures of ID when studying the etiology of health inequalities, especially during childhood.

Although this study had many strengths, several limitations should be considered. First, there are issues with the use of administrative claims data. The reliance on ICD-10 might yield some misclassification owing to the unavoidable characteristics of claims data, including miscoding, whether intended or not. However, efforts have been made by the government and hospitals, and currently, nearly 70% of primary, secondary, and tertiary diagnosis codes from claims records coincide with those from medical records in hospitals. Second, we could not include maternal information on diagnosis or treatment of thyroid disease, tobacco use, alcohol consumption, dietary habits, or other behavioral factors during pregnancy because of confidentiality. Furthermore, we could not ascertain the procedures performed or the health status of the newborns during/after birth because the database did not include complete information to ensure confidentiality. Therefore, some participants with incomplete information might be categorized with no disease status. Finally, our study results might have been influenced by unmeasured variables, as is the case with all observational studies. While we tried our best to identify and account for potential confounders, such efforts are inherently imperfect.

## Conclusion

This study identified associations among NH, low family income, and the risk of ID. Moreover, NH and low family income were found to significantly increase the risk of ID. These findings accentuate the importance of accurate detection programs for hypothyroidism in newborns. Public health policymakers should consider providing additional resources for alleviating NH for low-income families.

## Supporting information

S1 Table Combined effect of hypothyroidism and household income on intellectual disability. Adjusted for sex, residence area, low birth weight, birth asphyxia, congenital malformations, chromosomal abnormalities, and inborn errors of metabolism.
HR, hazard ratio; CI, confidence interval; ID, intellectual disability; HT, hypothyroidism; NHT, no hypothyroidism; INC, household income.
(DOCX)

S2 Table General characteristics of participants excluding Down syndrome by hypothyroidism and household income during 2002–2011. (DOCX)

S3 Table Results of the Cox proportional hazards analysis: association between occurrence of intellectual disability and risk factors excluding Down syndrome. * Congenital malformations of the nervous system, eye, ear face and neck, the circulatory system, the respiratory system, genital organs, the urinary system, Cleft lip and cleft palate, Other congenital malformations of the digestive, Congenital malformations and deformations of the musculoskeletal system, Other congenital malformations.
(DOCX)

S4 Table Combined effect of neonatal hypothyroidism and family income on intellectual disability excluding Down syndrome. Adjusted for sex, residence area, low birth weight, birth asphyxia, congenital malformations, chromosomal abnormalities, and inborn errors of metabolism.
ID, intellectual disability; NH, neonatal hypothyroidism; No NH, not neonatal hypothyroidism.
*P < 0.05, **P < 0.01, ***P < 0.001.
†reference.
(DOCX)

## Abbreviations

ID: intellectual disability
CHT: congenital hypothyroidism
NH: neonatal hypothyroidism
TSH: thyroid-stimulating hormone
SES: socioeconomic status
NHIS-NSC: the National Health Insurance Service-National Sample Cohort
NHIS: National Health Insurance Service
NHI: National Health Insurance
ICD: International Classification of Diseases
HR: hazard ratio
CI: confidence interval
