# Supplementary material for: The effect of neonatal hypothyroidism and low family income on intellectual disability: A population-based cohort study
Source: PLoS One. 2018 Nov 7;13(11):e0205955. doi: 10.1371/journal.pone.0205955 (PMC6221285; doi:10.1371/journal.pone.0205955)
Supplement: S1 Table — Adjusted for sex, residence area, low birth weight, birth asphyxia, congenital malformations, chromosomal abnormalities, and inborn errors of metabolism. HR, hazard ratio; CI, confidence interval; ID, intellectual disability; HT, hypothyroidism; NHT, no hypothyroidism; INC, household income. (DOCX) [file pone.0205955.s001.docx]

**S1 Table. Combined effect of hypothyroidism and household income on intellectual disability**

|  |  | **Intellectual disability** | | |  |
| --- | --- | --- | --- | --- | --- |
|  |  | **HR** | **95% CI** | | **P-value** |
| **Combination** | |  |  |  |  |
|  | HT × low INC | 36.05 | (12.79- | 101.61) | <.0001 |
|  | HT × middle INC | 5.50 | (1.82- | 16.61) | 0.0025 |
|  | HT × high INC | 2.46 | (0.56- | 10.82) | 0.2348 |
|  | NHT × low INC | 2.11 | (1.46- | 3.07) | <.0001 |
|  | NHT × middle INC | 1.19 | (0.83- | 1.71) | 0.3488 |
|  | NHT × high INC | 1.00 |  |  |  |

Adjusted for sex, residence area, low birth weight, birth asphyxia, congenital malformations, chromosomal abnormalities, and inborn errors of metabolism.

HR, hazard ratio; CI, confidence interval; ID, intellectual disability; HT, hypothyroidism; NHT, no hypothyroidism; INC, household income
