# Supplementary material for: The effect of neonatal hypothyroidism and low family income on intellectual disability: A population-based cohort study
Source: PLoS One. 2018 Nov 7;13(11):e0205955. doi: 10.1371/journal.pone.0205955 (PMC6221285; doi:10.1371/journal.pone.0205955)
Supplement: S2 Table — (DOCX) [file pone.0205955.s002.docx]

| **S2 Table. General characteristics of participants excluding Down syndrome by hypothyroidism and household income during 2002-2011** | | | | | | | | | | | | | | | |
| --- | --- | --- | --- | --- | --- | --- | --- | --- | --- | --- | --- | --- | --- | --- | --- |
|  | | **Total** | |  | **Hypothyroidism** | | | |  | **Household income** | | | | | |
|  |  |  |  |  | **Yes (n = 115)** | | **No (n = 91088)** | |  | **Low (n = 19927)** | | **Middle (n = 39135)** | | **High (n = 32141)** | |
|  |  | N | (%) |  | N | (%) | N | (%) |  | N | (%) | N | (%) | N | (%) |
| **Intellectual disability** | | |  |  |  |  |  |  |  |  |  |  |  |  |  |
|  | **No** | 91024 | (99.80) |  | 111 | (96.52) | 90913 | (99.81) |  | 19860 | (99.66) | 39065 | (99.82) | 32099 | (99.87) |
|  | **Yes** | 179 | (0.20) |  | 4 | (3.48) | 175 | (0.19) |  | 67 | (0.34) | 70 | (0.18) | 42 | (0.13) |
| **Hypothyroidism** | |  |  |  |  |  |  |  |  |  |  |  |  |  |  |
|  | No | 91088 | (99.87) |  | - |  | - |  |  | 19909 | (99.91) | 39085 | (99.87) | 32094 | (99.85) |
|  | Yes | 115 | (0.13) |  | - |  | - |  |  | 18 | (0.09) | 50 | (0.13) | 47 | (0.15) |
| **Household income** | |  |  |  |  |  |  |  |  |  |  |  |  |  |  |
|  | Q1(Low) | 19927 | (21.85) |  | 18 | (15.65) | 19909 | (21.86) |  | - |  | - |  | - |  |
|  | Q2 | 39135 | (42.91) |  | 50 | (43.48) | 39085 | (42.91) |  | - |  | - |  | - |  |
|  | Q3(High) | 32141 | (35.24) |  | 47 | (40.87) | 32094 | (35.23) |  | - |  | - |  | - |  |
| **Sex** |  |  |  |  |  |  |  |  |  |  |  |  |  |  |  |
|  | Man | 47188 | (51.74) |  | 48 | (41.74) | 47140 | (51.75) |  | 10267 | (51.52) | 20221 | (51.67) | 16700 | (51.96) |
|  | Women | 44015 | (48.26) |  | 67 | (58.26) | 43948 | (48.25) |  | 9660 | (48.48) | 18914 | (48.33) | 15441 | (48.04) |
| **Residence** | |  |  |  |  |  |  |  |  |  |  |  |  |  |  |
|  | Rural | 27194 | (29.82) |  | 25 | (21.74) | 27169 | (29.83) |  | 7308 | (36.67) | 11923 | (30.47) | 7963 | (24.78) |
|  | Urban | 64009 | (70.18) |  | 90 | (78.26) | 63919 | (70.17) |  | 12619 | (63.33) | 27212 | (69.53) | 24178 | (75.22) |
| **Low birth weight(<2500g)** | | |  |  |  |  |  |  |  |  |  |  |  |  |  |
|  | No | 90017 | (98.70) |  | 94 | (81.74) | 89923 | (98.72) |  | 19643 | (98.57) | 38659 | (98.78) | 31715 | (98.67) |
|  | Yes | 1186 | (1.30) |  | 21 | (18.26) | 1165 | (1.28) |  | 284 | (1.43) | 476 | (1.22) | 426 | (1.33) |
| **Birth asphyxia** | |  |  |  |  |  |  |  |  |  |  |  |  |  |  |
|  | No | 91091 | (99.88) |  | 114 | (99.13) | 90977 | (99.88) |  | 19895 | (99.84) | 39090 | (99.89) | 32106 | (99.89) |
|  | Yes | 112 | (0.12) |  | 1 | (0.87) | 111 | (0.12) |  | 32 | (0.16) | 45 | (0.11) | 35 | (0.11) |
| **Congenital malformations** | | |  |  |  |  |  |  |  |  |  |  |  |  |  |
|  | 0 | 80586 | (88.36) |  | 86 | (74.78) | 80500 | (88.38) |  | 17678 | (88.71) | 34620 | (88.46) | 28288 | (88.01) |
|  | 1 | 9550 | (10.47) |  | 20 | (17.39) | 9530 | (10.46) |  | 2015 | (10.11) | 4071 | (10.40) | 3464 | (10.78) |
|  | 2+ | 1067 | (1.17) |  | 9 | (7.83) | 1058 | (1.16) |  | 234 | (1.17) | 444 | (1.13) | 389 | (1.21) |
| **Chromosomal abnormalities** | | |  |  |  |  |  |  |  |  |  |  |  |  |  |
|  | No | 91080 | (99.87) |  | 112 | (97.39) | 90968 | (99.47) |  | 19908 | (99.90) | 39074 | (99.84) | 32098 | (99.87) |
|  | Yes | 123 | (0.13) |  | 3 | (2.61) | 120 | (0.53) |  | 19 | (0.10) | 61 | (0.16) | 43 | (0.13) |
| **Inborn error of metabolism** | | |  |  |  |  |  |  |  |  |  |  |  |  |  |
|  | No | 90714 | (99.46) |  | 112 | (97.39) | 90602 | (96.77) |  | 19811 | (99.42) | 38932 | (99.48) | 31971 | (99.47) |
|  | Yes | 489 | (0.54) |  | 3 | (2.61) | 486 | (3.23) |  | 116 | (0.58) | 203 | (0.52) | 170 | (0.53) |
| **Year of birth** | |  |  |  |  |  |  |  |  |  |  |  |  |  |  |
|  | 2002 | 9556 | (10.48) |  | 17 | (14.78) | 9539 | (10.47) |  | 2232 | (11.20) | 4270 | (10.91) | 3054 | (9.50) |
|  | 2003 | 9432 | (10.34) |  | 7 | (6.09) | 9425 | (10.35) |  | 2069 | (10.38) | 4253 | (10.87) | 3110 | (9.68) |
|  | 2004 | 9317 | (10.22) |  | 7 | (6.09) | 9310 | (10.22) |  | 2046 | (10.27) | 4152 | (10.61) | 3119 | (9.70) |
|  | 2005 | 8551 | (9.38) |  | 9 | (7.83) | 8542 | (9.38) |  | 1908 | (9.57) | 3763 | (9.62) | 2880 | (8.96) |
|  | 2006 | 7870 | (8.63) |  | 7 | (6.09) | 7863 | (8.63) |  | 1694 | (8.50) | 3399 | (8.69) | 2777 | (8.64) |
|  | 2007 | 9761 | (10.70) |  | 10 | (8.70) | 9751 | (10.71) |  | 2150 | (10.79) | 4179 | (10.68) | 3432 | (10.68) |
|  | 2008 | 9385 | (10.29) |  | 17 | (14.78) | 9368 | (10.28) |  | 2039 | (10.23) | 3895 | (9.95) | 3451 | (10.74) |
|  | 2009 | 8614 | (9.44) |  | 17 | (14.78) | 8597 | (9.44) |  | 1796 | (9.01) | 3578 | (9.14) | 3240 | (10.08) |
|  | 2010 | 9029 | (9.90) |  | 8 | (6.96) | 9021 | (9.90) |  | 1885 | (9.46) | 3724 | (9.52) | 3420 | (10.64) |
|  | 2011 | 9688 | (10.62) |  | 16 | (13.91) | 9672 | (10.62) |  | 2108 | (10.58) | 3922 | (10.02) | 3658 | (11.38) |
