# Supplementary material for: The effect of neonatal hypothyroidism and low family income on intellectual disability: A population-based cohort study
Source: PLoS One. 2018 Nov 7;13(11):e0205955. doi: 10.1371/journal.pone.0205955 (PMC6221285; doi:10.1371/journal.pone.0205955)
Supplement: S3 Table — * Congenital malformations of the nervous system, eye, ear face and neck, the circulatory system, the respiratory system, genital organs, the urinary system, Cleft lip and cleft palate, Other congenital malformations of the digestive, Congenital malformations and deformations of the musculoskeletal system, Other congenital malformations. (DOCX) [file pone.0205955.s003.docx]

| **S3 Table. Results of the Cox proportional hazards analysis: association between occurrence of intellectual disability and risk factors excluding Down syndrome.** | | | | | | |
| --- | --- | --- | --- | --- | --- | --- |
|  |  |  | Intellectual disability | | | |
|  |  | Person-year | N | HR | 95% CI | |
| Hypothyroidism | |  |  |  |  |  |
|  | No | 711611 | 175 | 1.00 |  |  |
|  | Yes | 869 | 4 | 7.86 | (2.70- | 22.89) |
| Household Income | |  |  |  |  |  |
|  | Q1(Low) | 155803 | 67 | 2.39 | (1.62- | 3.52) |
|  | Q2 | 308222 | 70 | 1.22 | (0.83- | 1.80) |
|  | Q3(High) | 248455 | 42 | 1.00 |  |  |
| Sex |  |  |  |  |  |  |
|  | Man | 369132 | 125 | 1.93 | (1.40- | 2.66) |
|  | Women | 343348 | 54 | 1.00 |  |  |
| Residence |  |  |  |  |  |  |
|  | Rural | 212514 | 69 | 1.42 | (1.05- | 1.93) |
|  | Urban | 499967 | 110 | 1.00 |  |  |
| Low birth weight ( < 2500g) | |  |  |  |  |  |
|  | No | 704134 | 167 | 1.00 |  |  |
|  | Yes | 8541 | 12 | 2.72 | (1.43- | 5.16) |
| Birth asphyxia | |  |  |  |  |  |
|  | No | 711639 | 178 | 1.00 |  |  |
|  | Yes | 841 | 1 | 0.95 | (0.13- | 7.14) |
| Congenital malformations* | |  |  |  |  |  |
|  | 0 | 630130 | 99 | 1.00 |  |  |
|  | 1 | 74390 | 52 | 3.93 | (2.80- | 5.52) |
|  | 2+ | 7961 | 28 | 14.28 | (8.96- | 22.76) |
| Chromosomal abnormalities | |  |  |  |  |  |
|  | No | 711551 | 169 | 1.00 |  |  |
|  | Yes | 930 | 10 | 11.02 | (5.48- | 22.15) |
| Inborn error of metabolism | |  |  |  |  |  |
|  | No | 708429 | 174 | 1.00 |  |  |
|  | Yes | 4052 | 5 | 2.81 | (1.15- | 6.89) |
| Year of birth | |  |  |  |  |  |
|  | 2002 | 114476 | 45 | 1.00 |  |  |
|  | 2003 | 103674 | 30 | 0.78 | (0.48- | 1.25) |
|  | 2004 | 93120 | 29 | 0.78 | (0.48- | 1.26) |
|  | 2005 | 76894 | 23 | 0.68 | (0.40- | 1.15) |
|  | 2006 | 62951 | 15 | 0.59 | (0.32- | 1.07) |
|  | 2007 | 68328 | 16 | 0.61 | (0.34- | 1.12) |
|  | 2008 | 56316 | 13 | 0.74 | (0.38- | 1.42) |
|  | 2009 | 43068 | 4 | 0.34 | (0.12- | 0.99) |
|  | 2010 | 36112 | 4 | 0.67 | (0.23- | 2.00) |
|  | 2011 | 29064 | 0 | 0.00 | - |  |
| * Congenital malformations of the nervous system, eye, ear face and neck, the circulatory system, the respiratory system, genital organs, the urinary system, Cleft lip and cleft palate, Other congenital malformations of the digestive, Congenital malformations and deformations of the musculoskeletal system, Other congenital malformations | | | | | | |
