# Supplementary material for: The effect of neonatal hypothyroidism and low family income on intellectual disability: A population-based cohort study
Source: PLoS One. 2018 Nov 7;13(11):e0205955. doi: 10.1371/journal.pone.0205955 (PMC6221285; doi:10.1371/journal.pone.0205955)
Supplement: S4 Table — Adjusted for sex, residence area, low birth weight, birth asphyxia, congenital malformations, chromosomal abnormalities, and inborn errors of metabolism. ID, intellectual disability; NH, neonatal hypothyroidism; No NH, not neonatal hypothyroidism. *P < 0.05, **P < 0.01, ***P < 0.001. †reference. (DOCX) [file pone.0205955.s004.docx]

| **S4 Table. Combined effect of neonatal hypothyroidism and family income on intellectual disability excluding Down syndrome**   \|  \|  \| **Intellectual disability** \| \| \| \| --- \| --- \| --- \| --- \| --- \| \|  \|  \| **HR** \| **95% CI** \| \| \| **Combined** \| \|  \|  \|  \| \|  \| HT × low INC \| 72.97 \| (9.93- \| 536.24) \| \|  \| HT × middle INC \| 9.29 \| (2.65- \| 32.53) \| \|  \| HT × high INC \|  \|  \|  \| \|  \| NHT × low INC \| 2.32 \| (1.57- \| 3.42) \| \|  \| NHT × middle INC \| 1.20 \| (0.81- \| 1.77) \| \|  \| NHT × high INC \| 1.00 \|  \|  \|   Adjusted for sex, residence area, low birth weight, birth asphyxia, congenital malformations, chromosomal abnormalities, and inborn errors of metabolism.  ID, intellectual disability; NH, neonatal hypothyroidism; No NH, not neonatal hypothyroidism  **P* < 0.05, ***P* < 0.01, ****P* < 0.001  †reference |
| --- | --- | --- | --- | --- | --- | --- | --- | --- | --- | --- | --- | --- | --- | --- | --- | --- | --- | --- | --- | --- | --- | --- | --- | --- | --- | --- | --- | --- | --- | --- | --- | --- | --- | --- | --- | --- | --- | --- | --- | --- | --- | --- | --- | --- | --- |
